# Antibacterial and Antibiofilm Activity of Layers Enriched with Silver Nanoparticles on Orthodontic Microimplants

**DOI:** 10.3390/jfb16030078

**Published:** 2025-02-22

**Authors:** Magdalena Sycińska-Dziarnowska, Magdalena Ziąbka, Katarzyna Cholewa-Kowalska, Karolina Klesiewicz, Gianrico Spagnuolo, Steven J. Lindauer, Hyo-Sang Park, Krzysztof Woźniak

**Affiliations:** 1 Department of Maxillofacial Orthopaedics and Orthodontics, Pomeranian Medical University in Szczecin, Al. Powst. Wlkp. 72, 70111 Szczecin, Poland; 2 Department of Ceramics and Refractories, Faculty of Materials Science and Ceramics, AGH University of Krakow, al. A. Mickiewicza 30, 30059 Krakow, Poland; 3 Department of Glass Technology and Amorphous Coatings, Faculty of Materials Science and Ceramics, AGH University of Krakow, 30059 Krakow, Poland; 4 Department of Pharmaceutical Microbiology, Faculty of Pharmacy, Jagiellonian University Medical College, 9 Medyczna St., 30688 Krakow, Poland; 5 Department of Neurosciences, Reproductive and Odontostomatological Sciences, University of Naples “Federico II”, 80131 Napoli, Italy; 6 School of Dentistry, College of Dental Medicine, Kaohsiung Medical University, Kaohsiung 80708, Taiwan; 7 Department of Orthodontics, School of Dentistry, Virginia Commonwealth University, Richmond, VA 23103, USA; 8 Department of Orthodontics, College of Dentistry, Kyungpook National University, Daegu 41940, Republic of Korea; parkhs@knu.ac.kr

**Keywords:** antibacterial function, bioactive coatings, orthodontic microimplants, silver nanoparticles

## Abstract

Orthodontic microimplants have revolutionized anchorage in orthodontics but remain vulnerable to microbial colonization, potentially leading to infection and failure. Surface modifications incorporating silver nanoparticles (AgNPs) offer antimicrobial benefits, providing long-term protection against bacterial infections, while improving partial osseointegration. This study investigates hybrid coatings enriched with AgNPs, calcium (Ca), and phosphorus (P) to improve antimicrobial efficacy and reduce biofilm formation. Microimplants fabricated from the Ti6Al4V alloy were divided into six groups with varying surface treatments, including etching in hydrofluoric acid and hybrid layers containing 0.5 mol% AgNPs and CaP. Antibacterial activity was evaluated using agar diffusion and biofilm formation assays against *S. aureus*, *E. coli*, and *S. mutans*. Surface roughness was analyzed and correlated with biofilm formation. The model assessing the impact of biomaterials on *S. aureus* biofilm revealed a strong association (R^2^ = 0.94), with biomaterial choice significantly influencing biofilm formation. The model for *E. coli* biofilm exhibited exceptional predictability (R^2^ = 0.99). The model for *S. mutans* biofilm demonstrated an association (R^2^ = 0.68). Hybrid coatings exhibited a promising antimicrobial activity. Biofilm formation was higher on microimplants with rougher surfaces. Hybrid coatings enriched with AgNPs and CaP enhance antimicrobial properties and partially reduce biofilm formation. It is suggested that the optimization of microimplant surface areas varies according to function. An enhanced performance can be achieved by maintaining a smooth surface for soft tissue contact, while incorporating a rough surface enriched with bactericidal and bioactive modifiers for bone contact areas.

## 1. Introduction

Orthodontic microimplants improve orthodontic treatment by providing reliable anchorage for complex tooth movements [1,2,3,4,5]. However, their success may be compromised by microbial colonization and subsequent infection, which can lead to complications or implant failure [6]. Meeting these challenges requires innovative surface modifications to enhance antimicrobial properties while maintaining biocompatibility and partial osseointegration. Partial osseointegration refers to the controlled integration of a microimplant into the surrounding bone tissue, where a deliberate and incomplete level of osseointegration is achieved.

The current gap in knowledge revolves around addressing the microbial challenges associated with orthodontic microimplants, necessitating innovative solutions to ensure effective microbial control. The success of orthodontic microimplants ranges between 85 and 95%, and factors affecting their failure were thought to be surface bone fracture, root contact, and mobility after inflammation or infection [6,7]. As the control of inflammation around microimplants is a crucial factor to reduce failure, innovative solutions are needed to ensure effective microbial control [6]. Using mouthrinses can assist in preventing inflammation and infection [8,9], and antibiotic use during implant surgery was found to be protective against complications in dental implant studies [10]. However, increasing antibiotic resistance among bacterial strains poses an additional concern [11]. Orthodontic treatment, while transformative for dental alignment, can also have implications for oral health, particularly because the presence of orthodontic appliances alters the bacterial flora. One noteworthy bacterium often associated with orthodontic procedures is *Streptococcus (S.) mutans* [12]. In addition, *Staphylococcus (S.) aureus* is a common bacterium that can cause various infections, including surgical site infections. Following implant surgery, the presence of *S. aureus* can lead to postoperative infection, jeopardizing the success of the procedure [13].

Current orthodontic microimplants may lack the optimal features to combat bacterial colonization, prompting exploration into materials like silver, which is known for its robust antimicrobial properties [14]. The surface modification of biomedical materials plays a crucial role in improving both osseointegration and resistance to bacterial infection [15]. The surface roughness of microimplants also creates niches that can promote bacterial colonization and biofilm formation, posing a significant risk of infection. These infections can compromise microimplant success. Consequently, achieving an optimal balance between surface roughness and minimizing bacterial adhesion remains a critical challenge in microimplant design. Studies on microimplants and implants have shown that the incorporation of silver nanoparticles effectively provided additional antimicrobial properties [16,17,18,19,20]. Microimplant and implant research has also studied the biocompatible properties of hydroxyapatite to improve mechanical stability and promote osteointegration [17,21]. Partial osseointegration offers a critical advantage in orthodontic applications by providing stable anchorage while facilitating microimplant removal [22]. Ideal modifications focus on enhancing the interaction between the microimplant surface and bone, minimizing bacterial colonization to reduce the risk of biofilm formation, while simultaneously promoting partial osseointegration.

This study introduces an innovative approach by incorporating silver nanoparticles into the surface of orthodontic microimplants. The key advancement lies in the post-firing process, during which the organic matrix on the microimplant surface transforms into a layer enriched with silver nanoparticles, calcium, and phosphorus. This unique surface composition facilitates the formation of an apatite layer, which not only enhances the antimicrobial properties of the microimplants but also promotes a favorable environment for partial osseointegration.

To address these challenges and capitalize on potential benefits, this study poses the following research question: how does the incorporation of hybrid coatings enriched with silver nanoparticles, calcium, and phosphorus onto orthodontic microimplants impact their antimicrobial efficacy in correlation with surface roughness and microstructure?

## 2. Materials and Methods

### 2.1. Study Groups

Microimplants, designed as Orthodontic Temporary Anchorage Device (TAD) systems with a self-drilling thread and a diameter of 1.4 mm, were fabricated from a Ti6Al4V alloy by Dentos (Dalseo-Gu, Daegu, Korea). A total of 174 microimplants were divided into distinct groups for various analyses: 6 samples (1 per group) were allocated for control and surface analysis, 6 specimens were used for reproducibility characterization, and 162 samples (54 per bacterial strain) were utilized for antimicrobial testing across three bacterial strains.

These microimplants, measuring 10 mm in length and 1.4 mm in diameter, are smaller than conventional dental implants and feature a notably rougher surface texture.

Microimplants can be characterized based on their material composition and surface treatments. The first type is the un-etched titanium alloy Ti-6Al-4V, referred to as Ti. When this titanium alloy is etched in hydrofluoric acid (HF Chempur, Piekary Śląskie, Poland), it is designated as Ti-E. Further modifications include covering the HF-etched titanium alloy with a hybrid layer containing 0.5 mol% AgNPs, which is labeled as Ti-E-Ag. The additional incorporation of calcium (Ca) and phosphorus (P) into this hybrid layer results in the Ti-E-Ag-CaP variant. Similarly, the un-etched titanium alloy coated with a hybrid layer containing 0.5 mol% AgNPs is termed Ti-Ag, while the addition of Ca and P to this un-etched AgNP-containing hybrid layer creates the Ti-Ag-CaP microimplant.

### 2.2. Microstructural and Surface Texture Analysis

The preparation of hybrid sols and the coating process for microimplants was described in detail in a previous study [23]. The microstructural and surface texture evaluation, as well as the elemental composition of coated and uncoated microimplants, were examined using scanning electron microscopy (SEM). Additionally, the surface topography of the microimplants was characterized through Confocal Microscopy and elaborated on in an earlier manuscript [23].

### 2.3. Evaluation of Antibacterial Activities

The antibacterial activity of the tested layers was evaluated against reference bacterial strains derived from the American Type Culture Collection. The study covered Gram-positive cocci—*S. aureus* ATCC 25923 and *S. mutans* ATCC 25175—as well as the Gram-negative rod *Escherichia (E.) coli* ATCC 25922.

The culture of *S. aureus* and *E. coli* was conducted on tryptic-soy agar (TSA, Oxoid) under aerobic conditions for 24 h, while *S. mutans* was cultured on Columbia agar with 5% sheep blood (Oxoid) under an atmosphere with 10% CO_2_ (Genbag CO_2_, bioMerieux, Marcy-l’Étoile, France).

Antimicrobial activity was assessed using the agar well diffusion method. First, a pure 24 h culture of examined bacterial strains was prepared. The bacterial suspension, based on the equivalent of 0.5 McFarland, was prepared in sterile saline solution (0.95% sterile NaCl; bioMerieux) from pure culture. The inoculum of *S. aureus* and *E. coli* was spread onto Mueller–Hinton agar plates (Oxoid), while the suspension of *S. mutans* was spread onto Mueller–Hinton agar plates with 5% sheep blood (Oxoid) using sterile cotton swabs. Subsequently, the well with a diameter of 8 mm was punched with a sterile agar punch tool and filled with 100 µL of liquid base solution for the hybrid layer. Then, plates were incubated at 37 °C for 24 h—*S. aureus* and *E. coli* were under aerobic conditions, while *S. mutans* was under an atmosphere with 10% CO_2_ (Genbag CO_2_, bioMerieux). The diameter of the inhibition zones around each well was measured after the incubation period. All tests were performed in triplicate.

### 2.4. Assessment of the Antibiofilm Properties

The antibiofilm activity was evaluated on six types of biomaterials, i.e., uncoated, etched, and coated with distinct layers, according to sample nomenclature.

Firstly, a suspension with a density of 5 × 10^5^ CFU/mL was prepared from a pure 24 h bacterial culture. The suspension was prepared in Mueller–Hinton broth (Oxoid) for *S. aureus* and *E. coli* strains, and in Brain Heart Infusion (BHI, Oxoid) broth supplemented with 5% fetal bovine serum (Sigma) for *S. mutans* strains. Subsequently, the investigated microimplants were placed in separate wells of a 12-well plate (Sarsted), with each microimplant in a separate well. Then, 2 mL of appropriately prepared microbial suspension was added to each well. The samples were then incubated for 24 h at 37 °C under aerobic conditions for *S. aureus* and *E. coli*, and in a CO_2_-enriched atmosphere for *S. mutans*.

After the incubation period, the biofilms of the tested samples were evaluated. The slightly modified Christensen method with crystal violet staining was adopted to evaluate the amount of biofilm formed on the microimplants [24]. Following the incubation period, the supernatant was decanted, and each microimplant was transferred to a well in a sterile 12-well plate and rinsed with PBS. The biofilm was dried at 60 °C for 30 min and subsequently stained with 0.1% crystal violet solution in PBS. After a 20 min incubation period with shaking, the excess dye was removed and the microimplants were washed again three times with PBS. Next, 1 mL of 96% ethanol was added to each well to dissolve the crystal violet absorbed by the biofilm. Then, from each well, 3 samples of 200 µL were transferred onto 96-well titrate plates. The absorbance was measured at 600 nm using a Tecan microplate reader (Biosan, Riga, Latvia). The positive control of biofilm formation for each tested bacterial strain and negative control of MH bullion were conducted. All tests were run in triplicate.

### 2.5. Scanning Electron Microscopy and Laser Confocal Microscope

The bacteria morphology after in vitro culturing on the surface of microimplants covered with hybrid layers was carried out by means of scanning electron microscopy (SEM; Apreo 2S Low vac, ThermoFisher Scientific, Waltham, MA, USA). The observations were carried out in high vacuum conditions using the ETD detector at the 15 kV accelerating voltage. For observation, all implants were stuck onto microscope stubs using carbon-conductive tape. The observations were carried out without sputtering the implant’s surface with a conductive layer.

The surface topography of microimplants was performed using a laser confocal microscope Lext OLS 4000 (Olympus, Tokyo, Japan) with a magnification of 50×. The scanned area for each measurement was 640 × 640 μm, providing a detailed representation of the microimplant surface.

### 2.6. Statistical Analysis

The research aimed to assess how biomaterials, treated as the independent variable, impact the formation of bacterial biofilms (*S. aureus*, *E. coli*, and *S. mutans*) and surface roughness. Four univariate regression models were constructed—one for each bacterial species and one for surface roughness. Each univariate regression model assesses the relationship between the biomaterial type (independent variable) and one of the four outcome variables. The models’ adequacy and explanatory power were measured using the coefficient of determination (R^2^), indicating the strength of the association between exposure and outcome. Differences in the outcome metrics corresponding to the array of biomaterials under investigation were rigorously quantified by evaluating the contrasts in the estimated marginal means (EMMs), which were derived from the fitted regression models. These EMMs represent the average expected outcome for each biomaterial in the model.

To statistically discern the magnitude and significance of the differences among the biomaterials, a methodical contrast analysis was employed. This analytical procedure facilitated a pairwise comparison between the EMMs of different biomaterials, thus enabling a nuanced assessment of their relative impacts on the outcome variables. The utilization of contrast analysis is instrumental in understanding which specific biomaterials differ significantly from each other and provides a basis for making informed conclusions about their comparative effectiveness.

For the contrast analysis, the Šidák correction method was applied to account for multiple comparisons. This included 15 pairwise comparisons among six groups for bacterial biofilm assessment and 10 pairwise comparisons among five groups for surface roughness evaluation. The application of the Šidák correction was intended to maintain the integrity of statistical significance by controlling the family-related error rate, given the multiple testing scenario. The magnitude of the observed differences was estimated using Cohen’s *d*, a standardized measure of effect size [25].

Both confidence intervals and *p*-values were computed using statistical approximations tailored to the specific models and applied methods. To achieve this, an approximation of the *t*-distribution was employed.

The results from the pairwise comparisons, which were based on general linear hypothesis testing, were visually represented using a compact letter display system. This method of presentation, as detailed by Hothorn et al. [26,27], allows us to discern which groups differ significantly from each other after adjusting for multiple comparisons.

The relationship between bacterial biofilm formation parameters and surface roughness was analyzed using Pearson’s correlation analysis. An asymptotic confidence interval for the correlation coefficient, obtained through Fisher’s transformation, was provided. *p*-values less than 0.05 were considered statistically significant. Analyses were conducted using the R Statistical language [28] on Windows 10 pro 64 bit (build 19045).

## 3. Results

**Figure 1:**
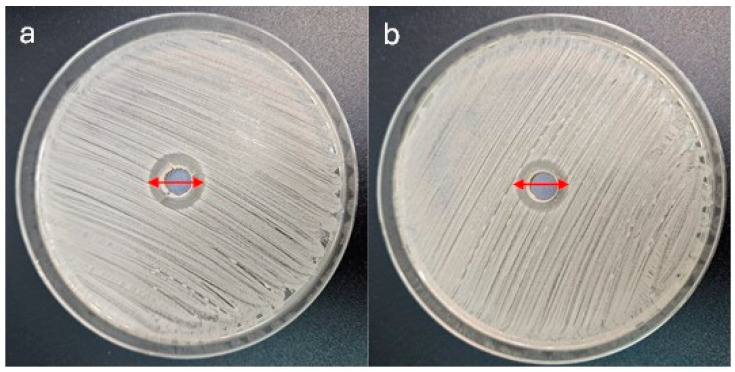
Activity of hybrid layers. (**a**) Containing only AgNPs and (**b**) enriched with CaP and AgNPs against *S. aureus* strain ATCC 25923 cultured for 24 h at 37 °C under aerobic conditions. The widest diameter of inhibition zone for AgNPs is 14mm; for CaP and AgNPs, this value is 13 mm.

Microbiological assays showed the good antibacterial proprieties of the base solutions. The diameter of the clear zones surrounding each well was measured in millimeters using a digital caliper. As is customary in such assays, the measurements were taken at the widest point of the inhibition zone. Each measurement was repeated in triplicate to ensure the consistency and reliability of the results. The zones of inhibition around the tested base solution for layers with AgNPs were as follows: 13.33 mm for *S. aureus* (Figure 1a); 12.67 mm for *E. coli*; and 10.67 mm for *S. mutans.* For layers enriched with CaP and AgNPs, these values were as follows: 12.33mm for *S. aureus* (Figure 1b); 11.67 mm for *E. coli*; and 19.67 mm for *S. mutans* (results from three repetitions).

**Figure 2:**
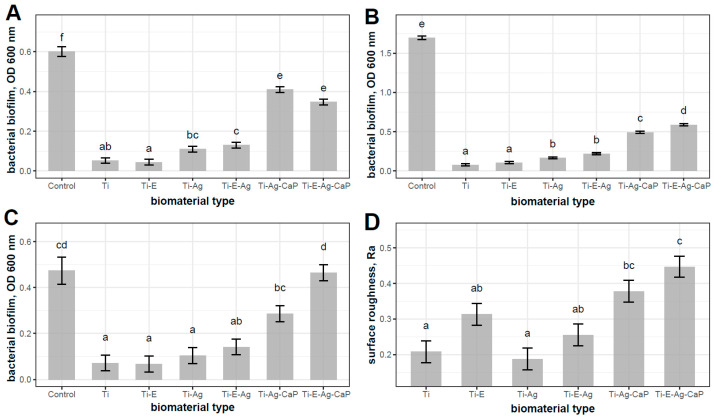
The EMMs with SE of the biomaterials studied and their effects on the bacterial biofilms of *S. aureus* (**A**), *E. coli* (**B**), *S. mutans* (**C**), and surface roughness (**D**), with a compact letter display for all pairwise comparisons. Statistically significant differences (*p* < 0.05) between the tested materials are marked a-f for different bacteria species.

A data set consisting of nine measurements for each of the six types of biomaterials and three measurements for the control group was carefully analyzed. This resulted in a total of 57 observations, providing an empirical basis for fitting regression models to reveal the effect of biomaterial type on bacterial biofilm formation (Figure 2). The model assessing the impact of biomaterials on *S. aureus* biofilm revealed a strong association (R^2^ = 0.94), with biomaterial choice significantly influencing biofilm formation. The model for *E. coli* biofilm exhibited exceptional predictability (R^2^ = 0.99). The model for *S. mutans* biofilm demonstrated an association (R^2^ = 0.68). The lowest amount of bacteria was observed for the specimen control (pure titanium microimplant) and etched microimplants (Ti, Ti-etched), as well as the control and etched microimplants with a layer containing AgNPs (Ti-E-Ag, Ti-Ag). Compared to the control microimplant (Ti), a statistically greater increase in bacterial biofilm was observed for layers containing AgNPs and CaP applied to the surface of both etched and un-etched microimplants (Ti-E-Ag-CaP, Ti-Ag-CaP). Additional results of statistical analysis were included in Supplementary Materials.

**Figure 3:**
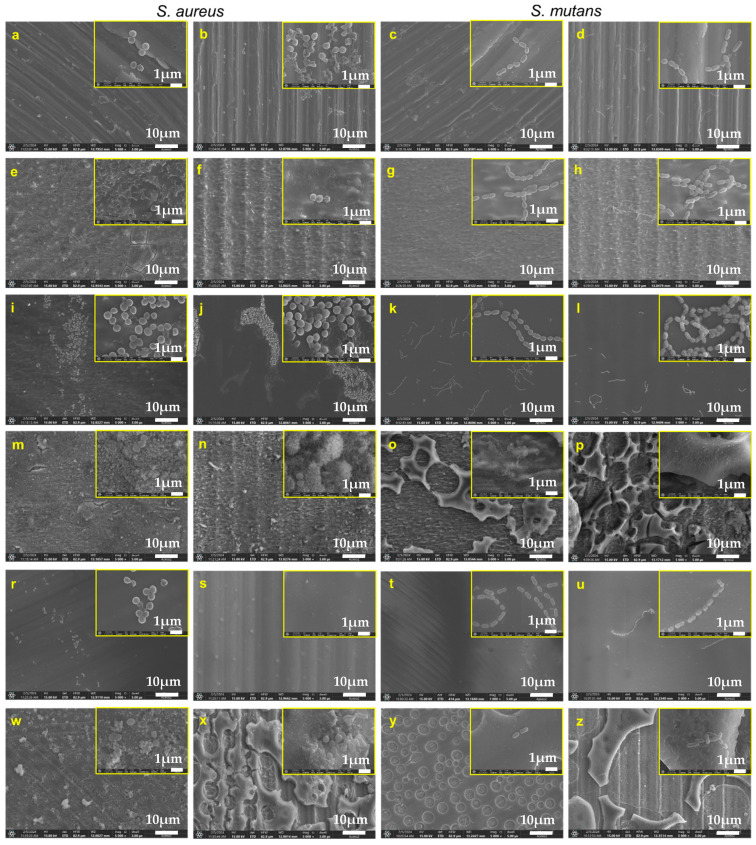
SEM images of *S. aureus* and *S. mutans* on the microimplant surfaces of Ti (**a**–**d**), Ti-etched (**e**–**h**), Ti-etched with layer with AgNPs (**i**–**l**), Ti-etched with layer with AgNPs and enriched with CaP (**m**–**p**), Ti with layer with AgNPs (**r**–**u**), and Ti with layer with AgNPs and enriched with CaP (**w**–**z**). The left-hand column of images presents the surface of the microimplant head; the right-hand column presents the microimplant thread. Magnification: 5000× and 50,000×.

Based on the observation of microimplant surfaces after biological testing, the dependence of the effect of microimplant roughness on the amount of bacterial biofilm was confirmed (Figure 3).

Table 1 summarizes the strong pairwise correlations in biofilm formation among *S. aureus*, *E. coli*, and *S. mutans*. Pearson’s correlation coefficients are notably high (exceeding 0.88), with correspondingly significant *p*-values, denoting a pronounced positive linear association between the biofilm formation propensities of these bacterial species under the studied conditions.

### Biofilm–Surface Roughness Relationships

In Table 2, correlations between bacterial biofilm formation and surface roughness reveal notable associations. *E. coli* and *S. mutans* show strong correlations, suggesting that smoother surfaces may reduce biofilm formation. Though not statistically significant for *S. aureus*, the trend implies a potential role for surface roughness.

In the case of all microimplants tested against all strains, it was noted that more bacteria were present on the thread of the microimplant compared to the surface of the head (right-hand columns in Figure 3). This is related to better microbial adhesion to surfaces with higher roughness and irregularities. The lowest amount of bacteria was observed on the surface of the Ti control microimplants (Figure 3a–d) and Ti-E etched microimplants (Figure 3e–h). The microscopic observations are consistent with the quantitative results. Significantly more bacteria were observed on the surface of etched and un-etched microimplants coated with a layer containing only AgNPs (Ti-E-Ag, Ti-Ag) (Figure 3i–l,r–u). In contrast, fewer bacteria were observed on the surface of etched and un-etched microimplants coated with a layer containing AgNPs and CaP (Ti-E-Ag-CaP, Ti-Ag-CaP), which does not correlate with the numerical data and is most likely related to the absorption of the crystal violet used to color the biofilm by the more porous layers.

## 4. Discussion

This study explores the surface modifications of Ti6Al4V microimplants to enhance their antimicrobial properties, focusing on the impact of etching and coating with layers containing silver nanoparticles and calcium phosphate. Layers containing silver nanoparticles demonstrated a superior ability to inhibit biofilm formation compared to coatings enriched with calcium and phosphorus. The microstructure, influenced by the technological process, etching, and layer porosity, significantly impacted biofilm formation. The porosity and surface characteristics of the coating affected how the bacteria could adhere to the microimplant, influencing the effectiveness of the antimicrobial performance.

To date, only three in vitro studies have explored silver nanoparticle layers on orthodontic microimplants [16,17,18]. Subramanian et al. reported on the antibacterial activity of Ti-BP-AgNPs against *Lactobacillus* and *S. aureus*, with weaker effects on *S. mutans* [16]. Venugopal et al. found no antimicrobial effect for Ti-AgNP-enriched microimplants initially but observed inhibition zones after 24 h [18]. A third study demonstrated the strong antibacterial activity of Ag/HA nanoparticles against *E. coli* and *E. faecalis*, with statistically significant reductions in bacterial colonies [17].

A comparative analysis with previous research indicates that minimally rough titanium microimplants exhibit higher residual bacteria than smooth microimplants [18], in agreement with the findings of the current study, in which control microimplants, those with etched surfaces (Ti, Ti-etched), and those with a layer containing AgNPs (Ti-E-Ag, Ti-Ag) exhibited the least bacterial presence. Conversely, a statistically significant increase in bacterial biofilm formation was observed for layers containing both AgNPs and CaP applied to the surface of both etched and un-etched microimplants (Ti-E-Ag-CaP, Ti-Ag-CaP) compared to the control microimplant (Ti). This may have been due to, and correlated with, the microimplant surface roughness and layer porosity. The higher the roughness and porosity, the easier it was for bacteria to adhere to the microimplant surface and, thus, multiply faster.

The presence of residual bacteria was significantly more prominent in minimally rough titanium microimplants compared to smooth microimplants (*p* < 0.0001) in a study involving *E. coli* strains [29]. In a recent study on dental implants, smooth and porous silver coatings showed inhibitory effects on *E. coli*, *S. aureus*, and *L. acidophilus*, with porous silver coatings showing good antibacterial properties [20]. These findings were consistent with prior studies emphasizing the impact of surface characteristics on microbial behavior [30,31,32,33].

In our study, the presence of fewer bacteria on the surface of both etched and un-etched microimplants coated with a layer containing AgNPs and CaP (Ti-E-Ag-CaP, Ti-Ag-CaP) appears inconsistent with the numerical data, suggesting a probable correlation with the absorption of the crystal violet used for biofilm staining by the more porous layers. Although the modified Christensen method with CV staining is a widely used and validated assay for biofilm quantification, it has some limitations, particularly when applied to porous surfaces. The greater porosity of some hybrid layers may have led to increased dye uptake, potentially overestimating biofilm biomass in some cases. Nevertheless, the use of complementary methods such as SEM imaging provided valuable qualitative insights that confirm the general trends observed in biofilm formation with different surface modifications. These findings seem particularly noteworthy due to the fact that layers containing AgNPs and CaP may have comparable antimicrobial properties to layers with AgNPs, while also having better bioactive properties to improve the osteointegration of the microimplant with bone. Moreover, the incorporation of both micro- and nano-scale roughness generates features that mirror the natural extracellular matrix structure. This configuration positively impacts cell functions by engaging with protein and cell membrane receptors across diverse scales [34].

The developed layers show antimicrobial potential, given the results obtained for the inhibition of biofilm formation, but an increased porosity of the layers, a higher roughness, and problems with the adhesion of the layers to microimplants of different portions; these are factors to be taken into account when designing the composition of the layers. Increasing the AgNP content above 0.5 mol % would likely provide more effective antimicrobial protection. On the other hand, a higher application speed would result in thinner and better adherent layers to the microimplants. The adhesion of coatings to microimplant surfaces remains a critical challenge, particularly for orthodontic microimplants subjected to complex biomechanical and environmental conditions. Factors such as surface preparation, coating thickness, and the compatibility between the coating materials play vital roles in determining adhesion strength. Hybrid coatings, which combine more components, offer potential advantages in this context by enhancing mechanical interlocking and chemical bonding with the substrate. In the current study, the hybrid coating design aims to improve adhesion by leveraging synergistic interactions between its components, ensuring a durable and stable layer under clinical conditions. Future investigations should focus on optimizing the preparation methods and conducting long-term adhesion tests to evaluate the performance of the hybrid coating under physiological stress, to better understand its clinical applicability.

This study has several key strengths, including the largest sample size tested in the field of microimplant coatings, the introduction of an innovative coating design, and a focus on addressing the critical need for effective microbial control in microimplants. Recognizing the significance of microbial control, the study explores the potential of developing microimplants with bioactive layers enriched with silver nanoparticles to reduce the risk of inflammation around the microimplants. However, it is necessary to increase the amount of silver nanoparticles and consider the roughness of the structure, which will undergo further investigation.

## 5. Conclusions

The conclusions drawn from these observations indicate that the created layers exhibit potential antimicrobial activity. Coatings containing silver nanoparticles demonstrated promising antibacterial activity against *S. aureus*, *E. coli*, and *S. mutans*, suggesting their efficacy in reducing bacterial colonization and biofilm formation. The dip-coating process requires optimization to increase the AgNP content and produce thinner, more adherent layers on the microimplant surface. Integrating calcium and phosphorus into the coating increases the roughness and porosity of the surface, which promotes the adhesion and proliferation of microorganisms. To solve this challenge, the selective surface treatment of the different areas of the microimplants can increase their effectiveness by maintaining a smooth, bactericidal surface on the outer part and a bactericidal but rougher surface in the bone contact area. These results underscore the importance of surface engineering in microimplant design, providing a pathway for improving their clinical reliability and long-term success in orthodontic applications.

## Figures and Tables

**Table 1:** Comparative analysis of pairwise associations in biofilm formation among bacterial species, *N_pairs_* = 6.

Pairs of Bacterial Biofilm Formation	*r*	*p*	CI 95%
*S. aureus* vs. *E. coli*	0.96	0.002	0.68–1.00
*S. aureus* vs. *S. mutans*	0.88	0.021	0.23–0.99
*E. coli* vs. *S. mutans*	0.97	0.001	0.76–1.00

*N_pairs_*—number of pairs; *r*—Pearson’s correlation coefficient; *p*—*p*-value of statistical test; CI 95%—confidence interval 95%.

**Table 2:** Correlation between biofilm formation and surface roughness in bacterial species—pairwise comparative assessment, *N_pairs_* = 6.

Pairs of Bacterial Biofilm Formation	*r*	*p*	CI 95%
*S. aureus* vs. surface roughness	0.79	0.062	−0.06–0.98
*E. coli* vs. surface roughness	0.87	0.025	0.19–0.99
*S. mutans* vs. surface roughness	0.87	0.025	0.19–0.99

*N_pairs_*—number of pairs; *r*—Pearson’s correlation coefficient, *p*—*p*-value of statistical test; CI 95%—confidence interval 95%.

## Data Availability

The original contributions presented in this study are included in the article and Supplementary Materials. Further inquiries can be directed to the corresponding author.

## Supplementary Materials

The following supporting information can be downloaded at: https://www.mdpi.com/article/10.3390/jfb16030078/s1. Table S1: EMMs for the formation of *S. aureus* biofilms (OD 600 nm) across studied biomaterial types, *df* = 50; Table S2: Results of the contrast analysis for the biofilm formation of *S. aureus* (OD 600 nm) with effect sizes, *df* = 50, σ^2^ = 0.04; Table S3: EMMs for the formation of *E. coli* biofilms (OD 600 nm) across studied biomaterial types, *df* = 50; Table S4: Results of the contrast analysis for the biofilm formation of *E. coli* (OD 600 nm) with effect sizes, *df* = 50, σ^2^ = 0.04; Table S5: EMMs for the formation of *S. mutans* biofilms (OD 600 nm) across studied biomaterial types, *df* = 50; Table S6: Results of the contrast analysis for the biofilm formation of *S. mutans* (OD 600 nm) with effect sizes, *df* = 50, σ^2^ = 0.10; Table S7: EMMs for the surface roughness (Ra) across studied biomaterial types *df* = 55; Table S8: Results of the contrast analysis for the surface roughness (Ra) with effect sizes *df* = 55, σ^2^ = 0.10.
