# Supplementary material for: Antibacterial and Antibiofilm Activity of Layers Enriched with Silver Nanoparticles on Orthodontic Microimplants
Source: J Funct Biomater. 2025 Feb 22;16(3):78. doi: 10.3390/jfb16030078 (PMC11942985; doi:10.3390/jfb16030078)
Supplement: Supplementary file 1 [file jfb-16-00078-s001.zip › jfb-3448046-supplementary.pdf]

Supplementary information - results of the contrast analysis

Estimation the effect of biomaterials on the *S. aureus* bacterial biofilm

Table S1 EMMs for the formation of *S. aureus* biofilms (OD 600 nm) across studied biomaterial types, $df = 50$.

Biomaterial type	*EMM*	*SE*	*CI 95% ll*	*CI 95% ul*
**Control**	0.60	0.02	0.55	0.65
**Ti**	0.05	0.01	0.02	0.08
**Ti-E**	0.04	0.01	0.02	0.07
**Ti-E-Ag**	0.13	0.01	0.10	0.16
**Ti-E-Ag-CaP**	0.35	0.01	0.32	0.38
**Ti-Ag**	0.11	0.01	0.08	0.14
**Ti-Ag-CaP**	0.41	0.01	0.38	0.44

Table S2 Results of the contrast analysis for the biofilm formation of *S. aureus* (OD 600 nm) with effect sizes, $df = 50$, $\sigma^2 = 0.04$.

contrast	*estimate*	*SE*	*t*	*p*	*d*
**Control – Ti**	0.55	0.03	19.02	< **0.001**	12.68
**Control – Ti-E**	0.56	0.03	19.31	< **0.001**	12.87
**Control – Ti-E-Ag**	0.47	0.03	16.33	< **0.001**	10.89
**Control – Ti-E-Ag-CaP**	0.25	0.03	8.80	< **0.001**	5.87
**Control – Ti-Ag**	0.49	0.03	17.02	< **0.001**	11.34
**Control – Ti-Ag-CaP**	0.19	0.03	6.60	< **0.001**	4.40
**Ti – Ti-E**	0.01	0.02	0.41	1.000	0.20
**Ti – Ti-E-Ag**	-0.08	0.02	-3.80	**0.008**	-1.79
**Ti – Ti-E-Ag-CaP**	-0.29	0.02	-14.45	< **0.001**	-6.81
**Ti – Ti-Ag**	-0.06	0.02	-2.83	0.131	-1.33
**Ti – Ti-Ag-CaP**	-0.36	0.02	-17.56	< **0.001**	-8.28
**Ti-E – Ti-E-Ag**	-0.09	0.02	-4.21	**0.002**	-1.99
**Ti-E – Ti-E-Ag-CaP**	-0.30	0.02	-14.86	< **0.001**	-7.01
**Ti-E – Ti-Ag**	-0.07	0.02	-3.25	**0.043**	-1.53
**Ti-E – Ti-Ag-CaP**	-0.37	0.02	-17.97	< **0.001**	-8.47
**Ti-E-Ag – Ti-E-Ag-CaP**	-0.22	0.02	-10.65	< **0.001**	-5.02
**Ti-E-Ag – Ti-Ag-CaP**	-0.28	0.02	-13.76	< **0.001**	-6.49
**Ti-Ag – Ti-E-Ag**	-0.02	0.02	-0.97	1.000	-0.46
**Ti-Ag – Ti-E-Ag-CaP**	-0.24	0.02	-11.62	< **0.001**	-5.48
**Ti-Ag – Ti-Ag-CaP**	-0.30	0.02	-14.73	< **0.001**	-6.94
**Ti-Ag-CaP – Ti-E-Ag-CaP**	0.06	0.02	3.11	0.063	1.47

Estimation the effect of biomaterials on the *E. coli* bacterial biofilm

Table S3 EMMs for the formation of *E. coli* biofilms (OD 600 nm) across studied biomaterial types, *df* = 50.

Biomaterial type	*EMM*	*SE*	*CI 95% ll*	*CI 95% ul*
**Control**	1.70	0.02	1.66	1.74
**Ti**	0.08	0.01	0.05	0.10
**Ti-E**	0.11	0.01	0.08	0.13
**Ti-E-Ag**	0.22	0.01	0.19	0.24
**Ti-E-Ag-CaP**	0.59	0.01	0.56	0.61
**Ti-Ag**	0.17	0.01	0.14	0.19
**Ti-Ag-CaP**	0.49	0.01	0.47	0.52

Table S4 Results of the contrast analysis for the biofilm formation of *E. coli* (OD 600 nm) with effect sizes, *df* = 50, $\sigma^2 = 0.04$.

contrast	*estimate*	*SE*	*t*	*p*	*d*
**Control – Ti**	1.62	0.02	65.57	**< 0.001**	43.71
**Control – Ti-E**	1.59	0.02	64.41	**< 0.001**	42.94
**Control – Ti-E-Ag**	1.48	0.02	59.84	**< 0.001**	39.89
**Control – Ti-E-Ag-CaP**	1.11	0.02	44.91	**< 0.001**	29.94
**Control – Ti-Ag**	1.53	0.02	61.92	**< 0.001**	41.28
**Control – Ti-Ag-CaP**	1.21	0.02	48.80	**< 0.001**	32.53
**Ti – Ti-E**	-0.03	0.02	-1.63	0.913	-0.77
**Ti – Ti-E-Ag**	-0.14	0.02	-8.10	**< 0.001**	-3.82
**Ti – Ti-E-Ag-CaP**	-0.51	0.02	-29.21	**< 0.001**	-13.77
**Ti – Ti-Ag**	-0.09	0.02	-5.16	**< 0.001**	-2.43
**Ti – Ti-Ag-CaP**	-0.41	0.02	-23.71	**< 0.001**	-11.18
**Ti-E – Ti-E-Ag**	-0.11	0.02	-6.47	**< 0.001**	-3.05
**Ti-E – Ti-E-Ag-CaP**	-0.48	0.02	-27.59	**< 0.001**	-13.00
**Ti-E – Ti-Ag**	-0.06	0.02	-3.53	**0.019**	-1.67
**Ti-E – Ti-Ag-CaP**	-0.39	0.02	-22.08	**< 0.001**	-10.41
**Ti-E-Ag – Ti-E-Ag-CaP**	-0.37	0.02	-21.12	**< 0.001**	-9.95
**Ti-E-Ag – Ti-Ag-CaP**	-0.27	0.02	-15.61	**< 0.001**	-7.36
**Ti-Ag – Ti-E-Ag**	-0.05	0.02	-2.94	0.100	-1.38
**Ti-Ag – Ti-E-Ag-CaP**	-0.42	0.02	-24.05	**< 0.001**	-11.34
**Ti-Ag – Ti-Ag-CaP**	-0.32	0.02	-18.55	**< 0.001**	-8.74
**Ti-Ag-CaP – Ti-E-Ag-CaP**	-0.10	0.02	-5.50	**< 0.001**	-2.59

Estimation the effect of biomaterials on the *S. mutans* bacterial biofilm

Table S5 EMMs for the formation of *S. mutans* biofilms (OD 600 nm) across studied biomaterial types, *df* = 50.

Biomaterial type	*EMM*	*SE*	*CI 95% ll*	*CI 95% ul*
**Control**	0.47	0.06	0.35	0.59
**Ti**	0.07	0.03	0.00	0.14
**Ti-E**	0.07	0.03	0.00	0.14
**Ti-E-Ag**	0.14	0.03	0.07	0.21
**Ti-E-Ag-CaP**	0.46	0.03	0.39	0.53
**Ti-Ag**	0.10	0.03	0.03	0.17
**Ti-Ag-CaP**	0.29	0.03	0.22	0.36

Table S6 Results of the contrast analysis for the biofilm formation of *S. mutans* (OD 600 nm) with effect sizes, *df* = 50, $\sigma^2 = 0.10$.

contrast	*estimate*	*SE*	*t*	*p*	*d*
**Control – Ti**	0.40	0.07	5.81	< **0.001**	3.87
**Control – Ti-E**	0.41	0.07	5.88	< **0.001**	3.92
**Control – Ti-E-Ag**	0.33	0.07	4.80	< **0.001**	3.20
**Control – Ti-E-Ag-CaP**	0.01	0.07	0.13	1.000	0.09
**Control – Ti-Ag**	0.37	0.07	5.34	< **0.001**	3.56
**Control – Ti-Ag-CaP**	0.19	0.07	2.70	0.179	1.80
**Ti – Ti-E**	0.00	0.05	0.10	1.000	0.05
**Ti – Ti-E-Ag**	-0.07	0.05	-1.43	0.973	-0.68
**Ti – Ti-E-Ag-CaP**	-0.39	0.05	-8.03	< **0.001**	-3.79
**Ti – Ti-Ag**	-0.03	0.05	-0.66	1.000	-0.31
**Ti – Ti-Ag-CaP**	-0.21	0.05	-4.39	**0.001**	-2.07
**Ti-E – Ti-E-Ag**	-0.07	0.05	-1.53	0.948	-0.72
**Ti-E – Ti-E-Ag-CaP**	-0.40	0.05	-8.13	< **0.001**	-3.83
**Ti-E – Ti-Ag**	-0.04	0.05	-0.76	1.000	-0.36
**Ti-E – Ti-Ag-CaP**	-0.22	0.05	-4.50	**0.001**	-2.12
**Ti-E-Ag – Ti-E-Ag-CaP**	-0.32	0.05	-6.60	< **0.001**	-3.11
**Ti-E-Ag – Ti-Ag-CaP**	-0.14	0.05	-2.96	0.094	-1.40
**Ti-Ag – Ti-E-Ag**	-0.04	0.05	-0.77	1.000	-0.36
**Ti-Ag – Ti-E-Ag-CaP**	-0.36	0.05	-7.37	< **0.001**	-3.47
**Ti-Ag – Ti-Ag-CaP**	-0.18	0.05	-3.73	**0.010**	-1.76
**Ti-Ag-CaP – Ti-E-Ag-CaP**	-0.18	0.05	-3.64	**0.014**	-1.72

Estimation the effect of biomaterials on the surface roughness

Table S7 EMMs for the surface roughness (Ra) across studied biomaterial types *df* = 55.

Biomaterial type	*EMM*	*SE*	*CI 95% ll*	*CI 95% ul*
**Ti**	0.21	0.03	0.15	0.27
**Ti-E**	0.31	0.03	0.25	0.37
**Ti-E-Ag**	0.26	0.03	0.19	0.32
**Ti-E-Ag-CaP**	0.45	0.03	0.39	0.51
**Ti-Ag**	0.19	0.03	0.13	0.25
**Ti-Ag-CaP**	0.38	0.03	0.32	0.44

Table S8 Results of the contrast analysis for the surface roughness (Ra) with effect sizes *df* = 55, $\sigma^2 = 0.10$.

contrast	*estimate*	*SE*	*t*	*p*	*d*
**Ti – Ti-E**	-0.11	0.04	-2.45	0.234	-1.09
**Ti – Ti-E-Ag**	-0.05	0.04	-1.09	0.993	-0.49
**Ti – Ti-E-Ag-CaP**	-0.24	0.04	-5.68	< **0.001**	-2.48
**Ti – Ti-Ag**	0.02	0.04	0.48	1.000	0.21
**Ti – Ti-Ag-CaP**	-0.17	0.04	-3.94	**0.003**	-1.76
**Ti-E – Ti-E-Ag**	0.06	0.04	1.35	0.950	0.61
**Ti-E – Ti-E-Ag-CaP**	-0.13	0.04	-3.17	**0.036**	-1.39
**Ti-E – Ti-Ag**	0.13	0.04	2.92	0.073	1.31
**Ti-E – Ti-Ag-CaP**	-0.06	0.04	-1.50	0.897	-0.67
**Ti-Ag – Ti-E-Ag**	-0.07	0.04	-1.57	0.859	-0.70
**Ti-Ag – Ti-E-Ag-CaP**	-0.26	0.04	-6.16	< **0.001**	-2.69
**Ti-Ag – Ti-Ag-CaP**	-0.19	0.04	-4.42	**0.001**	-1.98
**Ti-E-Ag – Ti-E-Ag-CaP**	-0.19	0.04	-4.56	< **0.001**	-1.99
**Ti-E-Ag – Ti-Ag-CaP**	-0.12	0.04	-2.85	0.088	-1.27
**Ti-Ag-CaP – Ti-E-Ag-CaP**	-0.07	0.04	-1.64	0.815	-0.72
